# Views and preferences of food‐insecure pregnant women regarding food insecurity screening and support within routine antenatal care

**DOI:** 10.1111/hex.13956

**Published:** 2024-01-07

**Authors:** Julia Zinga, Paige van der Pligt, Fiona H. McKay

**Affiliations:** ^1^ School of Health and Social Development, Institute for Health Transformation, Faculty of Health Deakin University Melbourne Victoria Australia; ^2^ Department of Nutrition and Dietetics Royal Women's Hospital Parkville Victoria Australia; ^3^ School of Exercise and Nutrition Sciences, Institute for Physical Activity and Nutrition (IPAN) Deakin University Geelong Victoria Australia; ^4^ Department of Nutrition and Dietetics Western Health Footscray Victoria Australia

**Keywords:** food insecurity, patient preferences, pregnancy, screening

## Abstract

**Background:**

Food insecurity is a public health concern that has profound impact on physical and mental health, and on social well‐being. Pregnancy is a period in which food insecurity is likely to be particularly deleterious, due to the serious impact on both mother and child. Food insecurity is not routinely screened in antenatal healthcare settings, and the preferences of pregnant women regarding food insecurity screening and support are poorly understood. This study aimed to determine the views and preferences of food‐insecure pregnant women regarding food insecurity screening and support within antenatal healthcare.

**Methods:**

This qualitative descriptive study used face‐to‐face semi‐structured interviews, conducted in February and March 2023, to gain the views of purposively sampled food‐insecure, pregnant women in Melbourne, Australia. Food insecurity was evidenced by an affirmative response to at least one of three assessment items in a screening questionnaire. Qualitative content analysis was conducted to summarise the views and preferences of women.

**Results:**

Nineteen food‐insecure pregnant women were interviewed. Three themes were identified: (1) acceptability of being screened for food insecurity, (2) concerns about the consequences of disclosure and (3) preferences regarding food insecurity screening and supportive strategies that could be offered within an antenatal healthcare setting.

**Conclusion:**

Women were accepting of food insecurity screening being conducted within routine healthcare. Women identified potential benefits of routine screening, such as feeling supported by their clinician to have a healthy pregnancy and less pressure to voluntarily ask for food assistance. Women gave suggestions for the implementation of food insecurity screening to optimise their healthcare experience, maintain their dignity and feel able to disclose within a safe and caring environment. These results indicate that food insecurity screening in the antenatal setting is likely to have support from pregnant women and is urgently needed in the interest of promoting optimal nutrition for women and children.

**Patient Contribution:**

Pregnant women with lived experience of food insecurity were purposively sampled to obtain their insights regarding screening and support within a pregnancy healthcare setting. Member‐checking occurred following data collection, whereby all participants were offered the opportunity to review their interview transcript to ensure trustworthiness of the data.

## 1 INTRODUCTION

Food insecurity, the limited or uncertain availability of nutritionally adequate and safe foods, or limited or uncertain ability to acquire acceptable foods in socially acceptable ways,^1^ is a public health concern that has profound impact on physical and mental health, and on social well‐being.^2^ The burden of food insecurity can be felt by individuals across all life stages. Due to the serious and negative impact on both maternal and child health in women who are deemed food‐insecure, pregnancy is a period in which food insecurity is likely to be particularly deleterious. Food insecurity has been linked with impaired maternal mental health,^3^ inadequate and excess gestational weight gain,^4^ congenital abnormalities^5^ and preterm birth.^3^ This relationship is in part due to food insecurity contributing to disordered eating patterns induced by the stress of food scarcity^6^ and restricted access to a nutritionally adequate diet during pregnancy.^3^ Approximately 14% of Australian pregnant women experience food insecurity,^7^ with similar rates reported in other high‐income countries.^8^, ^9^

Food insecurity during pregnancy is influenced by a range of economic and social risk factors, the strongest predictor being income‐poverty.^10^ Women are more likely than men to experience poverty,^11^, ^12^ and thus, disproportionately bear the burden of food insecurity.^13^ The financial and social conditions that underpin food insecurity persist during pregnancy as women are more likely than men to be employed in lower‐wage roles,^12^, ^14^ particularly when they become mothers,^14^ and forego their food intake in favour of the nutritional needs of other household members.^15^, ^16^, ^17^ Alongside income‐poverty, other risk factors for food insecurity relevant to this population group are low maternal age, low education attainment, single‐parent households, ethnicity and acculturation.^10^, ^18^ The wide range of risk factors is indicative of the complex and multifactorial nature of food insecurity^19^ and emphasises the difficulty in delivering relief strategies to this priority population group.

The growing evidence base regarding the impact of food insecurity during pregnancy^3^ has propelled advocacy for food insecurity screening and interventions within antenatal healthcare.^20^, ^21^ For instance, the position of the American College of Obstetricians and Gynaecologists is that clinicians should assess, document and address ‘social and structural determinants of health that may influence a patient's health … such as access to food’.^20^ Nurses have similarly been called to action to advance health equity for women by addressing social determinants of health within routine healthcare.^22^ Despite these calls, food insecurity screening is not routinely conducted during antenatal healthcare.^23^ A recent systematic review found seven published studies investigating screening procedures within the antenatal healthcare setting, comprising four screening tools, with varying methods of implementation and minimal evaluation of effectiveness.^24^ There are also few interventions that address food insecurity during pregnancy within healthcare settings, beyond the screening process.^25^, ^26^, ^27^

Government‐level food assistance programmes have been established in some high‐income countries, to improve dietary quality of priority groups such as pregnant and postpartum women. The Special Supplemental Nutrition Program for women, infants and children (WIC) in the United States^28^ and the Healthy Start program in the United Kingdom^29^ provide access to nutritious foods and micronutrient supplements to low‐income pregnant women who have been deemed to be at nutritional risk. The Canada Prenatal Nutrition Program provides funding for community resources that promote the health of pregnant women, including micronutrient supplements and nutrition education.^30^ No nutrition assistance programmes for antenatal health promotion exist in Australia. Although there is good evidence to suggest that these programmes are effective at promoting dietary quality and food security,^31^, ^32^ uptake of WIC by eligible women is declining,^33^ and below target for the Healthy Start programme.^34^, ^35^ Cited reasons for low uptake include administrative burdens for participants,^34^ misconceptions about eligibility^36^ and stigma.^37^

Interventions that are ‘internally’ based in a healthcare setting are considered a viable option to screen and address food insecurity, to complement a referral to these established ‘external’ food assistance programmes.^27^ Healthcare‐based interventions are considered valuable by clinicians as well as patients, based on research in general, nonpregnancy healthcare settings.^25^, ^38^, ^39^, ^40^, ^41^ However, the acceptability and preferences of pregnant women regarding food insecurity screening and support within antenatal healthcare are poorly understood.^27^ Pregnant women have also been inadequately consulted to develop or evaluate government‐level food assistance programmes.^42^ The lack of inclusion of women's views is a crucial evidence gap that must be addressed before the development of screening and support processes that are effective, yet sensitive to the needs of this population group. Although research in nonpregnant food‐insecure adults suggests that patients perceive it appropriate for food insecurity to be screened during routine healthcare,^43^, ^44^ there is evidence that screening may inadvertently cause harm when implemented poorly.^45^, ^46^ The unique circumstances of pregnancy, a transitory situation but one that precipitates the construction of maternal identity that redefines a woman's self‐perception,^47^ mean that disclosure of food insecurity may be affected. The views of food‐insecure pregnant women regarding screening and support are required for an informed approach to future screening and possible intervention. Furthermore, given the lack of Australian nutrition assistance programmes for antenatal health promotion, the views of Australian women are particularly warranted before the potential establishment of new services for specific implementation within antenatal healthcare settings. This study aimed to determine the views and preferences of food‐insecure pregnant women attending a large maternity hospital in Melbourne, Australia, regarding food insecurity screening and support within routine antenatal healthcare.

## 2 METHODS

### 2.1 Study design

This qualitative descriptive study used face‐to‐face semi‐structured interviews to gain the views of purposively sampled food‐insecure, pregnant women in Melbourne, Australia, between February and March 2023. Qualitative descriptive studies provide a ‘straight description’ of the phenomenon,^48^ meaning that researchers stay close to the data in the analytical process.^49^ Although description is the aim of these studies, interpretation is also present and, thus, influenced by the perceptions and sensitivities of the researcher.^50^ Qualitative descriptive studies are an appropriate methodology to gain insights from informants regarding a poorly understood phenomenon,^48^ to answer questions about human behaviour, views and barriers^50^ and are considered particularly suited to health sciences research.^51^ The consolidated reporting criteria for qualitative studies were used to design and report this study.^52^ Ethics approval was granted by Royal Women's Hospital (RWH) Hospital HREC (02773/22‐33) and Deakin University HREC (2023‐016).

### 2.2 Recruitment and participants

Recruitment occurred at the Royal Women's Hospital, one of Australia's largest maternity hospitals with over 8000 annual births and a culturally diverse patient population that is representative of over 190 world nations.^53^ The first author is employed at the hospital as a clinical dietitian to provide nutritional care to pregnant women. Recruitment occurred in three ways: via advertising flyers, directly approaching women and social media advertisements. Advertising flyers, accessible in clinic rooms and patient waiting areas, featured a QR code linked to a screening questionnaire that was administered either online using Qualtrics software^54^ or in paper format. The screening questionnaire and advertising flyers were available in the five languages most commonly spoken by women attending the hospital (English, Hindi, Arabic, Vietnamese, Chinese).^55^ For direct recruitment, the first author invited pregnant women, who were at varying stages of gestation, to complete the questionnaire in paper format. Direct approach to all pregnant women waiting for their appointments in the hospital antenatal clinics occurred over six, 4‐h pregnancy outpatient clinics during February 2023. Lastly, recruitment occurred via one social media post about the study, embedded with an internet link to the screening questionnaire, and uploaded on hospital‐managed social media platforms (Facebook and Instagram).

The 27‐item eligibility screening questionnaire featured demographic questions relating to country of birth, household income, receipt of government welfare payments, current or previous use of food aid and three questions to assess food insecurity that were previously piloted with Australian pregnant women.^7^ The food insecurity assessment items were as follows: ‘I worried whether my/our food would run out before I/we got money to buy more’, ‘The food that I/we bought just didn't last, and I/we didn't have money to get more’ and ‘I/we couldn't afford to eat balanced meals’, relating to the previous 12 months. Response options were ‘often true’, ‘sometimes true’, ‘never true’ or ‘don't know.; a response of ‘often true’ or ‘sometimes true’ was considered to be an affirmative response.^7^ The final questionnaire item invited respondents to register their interest in participating in an individual interview by providing their name and phone number.

Eligible participants were pregnant women attending RWH for antenatal care who were experiencing food insecurity, evidenced by an affirmative response to at least one of the three food insecurity questions, or were currently or previously using food aid. Eligible participants who provided their name and phone number were contacted by the first author to discuss the study in more detail and obtain their email address to issue an additional plain language statement for informed consent to an interview. Recruitment continued until sufficient conceptual depth was obtained from the participants' accounts for a rich network of concepts and themes to be developed.^56^

### 2.3 Data collection

Interview guides used in qualitative descriptive studies tend to be more structured than in other qualitative methodologies, due to being typically based on expert knowledge to focus on areas that are poorly understood in a healthcare context and/or amenable to intervention.^50^ Thus, the interview guide for the present study was developed following the review of existing literature relating to views towards food insecurity screening in nonpregnant populations^57^, ^58^, ^59^, ^60^ and pregnant women's views about other antenatal screening processes.^61^ Questions aimed to gain insights into enablers and barriers to women's disclosure of food insecurity, as well as preferences for subsequent support strategies that could be offered by the hospital to address food insecurity. The interview guide was piloted with one pregnant woman experiencing food insecurity who was not a RWH patient (who was also compensated for her time); minor revisions to question sequencing were subsequently made (see the Supporting Information material for the interview guide).

In‐depth one‐on‐one interviews were conducted by the first author (an Australian‐born, food‐secure, dietitian and mother) over the phone, via videoconferencing or face‐to‐face, based on participant preference. In‐depth interviews are a key data collection method used in qualitative studies to gather data on participants' views and feelings about specific topics.^48^, ^50^, ^62^ Participants were aware that the interviewer (the first author) was employed at RWH as a clinical dietitian; however, they were not receiving consultative healthcare from the interviewer. A reflexive approach to data collection^63^ was used by the interviewer, whereby nonjudgemental curiosity and active listening enabled participants to comfortably share their insights. The potential for participants to become distressed during the interview was recognised and addressed by the development of a distress protocol. Additionally, the participant information and consent form forewarned participants about possible distress and included potential management strategies such as halting the interview, referral to internal counselling services and locally available food aid. A $AUD40 gift card was sent to participants after the interview in acknowledgement of their time and contribution.^64^ Interviews were audio‐recorded and transcribed verbatim by an external transcription company. All participants were offered the opportunity to review their interview transcript; three participants accepted, and no amendments to their individual responses were made.

### 2.4 Data analysis

Qualitative content analysis was conducted by the first author to summarise participants' information.^49^, ^50^ The interview guide served as an initial organising framework for deductive data analysis, with concepts also inductively derived from the interview transcripts. This reflexive process of modifying the treatment of data as new insights arise is a feature of qualitative content analysis, whereby pre‐existing coding systems may begin data analysis but they are modified to ensure best fit to the data.^49^ Data analysis, guided by the approach set out by Miles et al.,^62^ commenced with data immersion; transcripts were read and reread to become familiar with the data. Portions of text from the interview transcripts were then coded according to the main areas of interest to answer the research questions, developed a priori to data collection. Regularly occurring concepts that were inductively derived from the data were also coded.^62^ This resulted in a coding manual that was revised iteratively as data collection and analysis proceeded, and then used to recode previously coded data, in a constant comparative approach.^65^ Second‐cycle coding was then conducted to reorganise and reanalyse data, for the development of a coherent metasynthesis of the data.^62^ Trustworthiness and credibility of the analysis were enhanced through strategies such as taking field notes during interviews and memo‐writing. NVivo software^66^ was used for data management and coding.

## 3 RESULTS

Of the 295 women who completed the screening questionnaire, 97 were eligible for the study and 19 consented to be interviewed. Interviews were on average 32 min in length (range: 14–81 min). Sixteen participants completed the paper version of the screening questionnaire, two participants completed the survey online after taking a flyer and one participant responded to the social media advertisement for the study. The mean age of the participants was 32.8 years and ranged from 19 to 42 years. Nine responded affirmatively to all three food insecurity screening items, seven participants gave only one affirmative response. Most participants (*n* = 11) identified as culturally diverse/migrant from a range of low‐ to high‐income countries, and most (*n* = 12) were multiparous (had previously given birth to at least one child). Household income varied, with the same number of participants categorised as earning over $AUD120,000/year (*n* = 5) as participants categorised as earning $AUD20,000–$50,000/year (*n* = 5); two participants earned less than $AUD20,000/year. Table 1 describes participants' demographic characteristics.

**Table 1:** Participant characteristics.

Characteristic	*n* (%)
Gestational age	
Trimester 1 (<13 weeks)	0
Trimester 2 (13–26 weeks)	9 (47)
Trimester 3 (>26 weeks)	10 (53)
Number of children at home	
0	7 (37)
1	4 (21)
2	4 (21)
3	2 (11)
4	2 (11)
Household annual gross income (AUD)	
$0–$20,000	3 (16)
$21,000–$50,000	5 (26)
$51,000–$70,000	4 (21)
$71,000–$90,000	0
$91,000–$120,000	1 (5)
>$120,000	5 (26)
Ethnicity^a^	
Australian/Oceaniana^b^	8
South Asian	3
Middle Eastern	3
African	2
Southeastern European	1
North American	1
Southeast Asian	1
Government welfare recipient	
Yes	10 (53)
No	9 (47)
Food aid recipient (current or previous)	
Yes	7 (37)
No	12 (63)

^a^ Australian/Oceanian comprises Australia (Indigenous and non‐Indigenous), New Zealand and nearby Pacific Islands.

^b^ Based on country of birth response in the questionnaire and self‐reported cultural background in the interview.

Three themes were identified through analysis of the interviews. These themes relate to (1) the acceptability of being screened for food insecurity, (2) concerns about the consequences of disclosure and (3) the preferences regarding food insecurity screening and supportive strategies that could be offered within an antenatal healthcare setting.

1. *Acceptability of being screened for food insecurity*
  Food insecurity screening was considered an appropriate component of antenatal healthcare for all participants. Several factors influenced participants' acceptability of this process. These included ‘pragmatism’, ‘inferences of care and support’ and ‘inclusivity’.
  Participants held pragmatic views towards food insecurity screening, based on practical considerations for a healthy pregnancy. These views stemmed from an understanding of the need for nourishment where food was considered a ‘basic thing’ that ‘comes first in every sense’. Participants' awareness of the importance of antenatal nutrition was a factor in their views about food insecurity screening during antenatal healthcare.
  Because eating properly while you're pregnant is one of the most important things for development of the baby and brain development and stuff, so maybe there wouldn't be as much problems with babies if women were getting the right support. (40 years, first child, Australian)
  Such was their fundamental acceptance of food insecurity screening that participants considered screening an example of the routine surveillance to which pregnant women are commonly subjected. Food insecurity screening was conceptualised by participants as analogous to questions about mood, sleep and other pregnancy symptoms.
  I think it's just a pregnancy thing … you get asked so many random things when you're pregnant, when you're in hospital and with doctors. And it would just be another topic that just gets covered. (36 years, second child, Australian)
  Others went on to suggest that screening at other life stages may not be acceptable. Pregnancy was viewed by participants as being an exceptional time where sensitive topics could be discussed within the context of optimising well‐being.
  I say it's appropriate because of, if I'm not pregnant, I don't need somebody to ask these questions. But if I'm pregnant, you don't know my situation, what's going on. So maybe to ask this question, like, are you safe? Or about your food. I think that's appropriate. Both questions are appropriate. (34 years, third child, South Asian)
  Participants commented that maternal instinct to care for their baby, via an improved access to nourishing food, drove this change in attitude about screening acceptability during pregnancy. This was particularly true for multiparous participants.
  I think I've learned to drop my pride and ego a bit since becoming a mum. Before I'd say no, I can do things on my own. I don't need anyone's help … I think now having, being a mum and now having another baby, I understand why women need the support. (29 years, second child, South Asian)
  Participants' practical consideration of screening meant that their concern of embarrassment in discussing food insecurity would be outweighed by the benefit of being supported, both for themselves and their baby. Participants predicted that a positive outcome of food insecurity screening would be worth the risk of feeling vulnerable by disclosure to antenatal clinicians.
  Yeah, it is hard but I think it just needs to be addressed … There's no way you can really beat around the bush with it. If you're struggling, you're struggling. (22 years, first child, Australian)
  The prospect of food insecurity assessment within the antenatal healthcare setting was an indicator to participants that their healthcare provider was caring and supportive. The screening was inferred as an example of compassionate, holistic healthcare, which participants highly valued.
  …it just tells them, okay, this person is caring for me or this person want to know how I'm feeling so they can express themself which is actually really good. There is nothing bad about it. (31 years, third child, African)
  Removing the pressure from women to instigate their own request for support was considered a way for antenatal clinicians to provide support for the burden of food insecurity. Participants were not comfortable advocating for their own health, preferring routine assessment.
  Yeah, it's not like I have to be seeking out help, I have to recognize I have a problem. They're just asking it anyway. (24 years, first child, Australian)
  Participants' acceptability of screening also drew from notions of inclusivity. Knowing that all pregnant women would be screened as part of routine healthcare was considered positive as this would help reduce the stigma associated with food insecurity.
  I think I would respond to that quite well … especially if I knew that context of it being asked to everyone, I would think that was a good thing. (39 years, second child, Australian)
  The feeling of inclusivity was also fostered by the knowledge that many other women were experiencing financial hardship that was considered societal and commonly experienced, which enabled disclosure.
  I think now I'm way more transparent because I think a lot more people need help and they're talking about it or amongst themselves. (31 years, third child, Australian)
2. *Concerns about the consequences of disclosure*
  Although all participants were supportive of food insecurity screening, this acceptance was accompanied by trepidation from some participants. Concerns about the consequences of disclosing food insecurity were centred on a fear of being judged by clinicians, and of domestic tension.
  Some participants were aware of the stigma associated with an inability to provide enough quality food for their family and were concerned that the identification of food insecurity would result in clinician's misjudgement and suboptimal quality of care.
  There's a big stigma on pregnant women as well, so that people will be like, oh, how she can look after the baby when she has it, if she can't look after, you know, stuff now. (40 years, first child, Australian)
  Other participants worried that disclosure of food insecurity could open further invasive enquiry and forced validation of their circumstances. These concerns stemmed from some participants' negative experiences when interacting with government welfare agencies. One participant feared that routine screening and identification could lead to her children being removed from her care, a concern based on her own childhood experience of out‐of‐home care.
  I'd be scared you're gonna—you know—if I had kids, do you know what I mean? … Questions like that scare mums, especially my mum. (19 years, first child, Australian)
  In addition to clinician misjudgement, participants were concerned that disclosure could instigate domestic tension if their partner reacted negatively. One participant anticipated that her partner would have wounded pride, as the main income earner, and another participant predicted that her husband would be angry to be portrayed as one that could not provide for the family.
  Nothing would stop me, except for the fear of my husband finding out. [That would] make a bad situation an even worse situation. (33 years, fifth child, Middle Eastern)
  For migrant participants, cultural norms in domestic relationships were drawn upon to anticipate the tension caused by food insecurity disclosure. One participant stated,
  Let's say, there wasn't enough money and you're not working, and the man is. You're never going to meet a cultural woman that's going to say that they don't have enough food, which would infer that it's their husband's fault. The next question they would ask is, what else did you say? And that's already leading to a fight. (38 years, third child, Southeastern European)
  The cultural lens on this issue was shared by another migrant participant, where the concern that disclosure of inability to provide food could lead to shame was considered potentially problematic for the relationship dynamics.
  Because some appointment, husband is there. If my husband will come, I'll say yes, yes. I will not say anything unusual of course, because I have to go back with him … He might ask ‘Why? I'm earning enough’. Yeah, I think so because in our culture, again that shame thing. (34 years, third child, Southeast Asian)
  However, these perceived cultural norms were not shared by all migrant participants.
  There is nothing to hide from the partner, that's fine with me. If my husband is there with me and then the midwife or the doctor or somebody is asking me this question, he will feel happy to even participate in it. (31 years, third child, African)
  Participants in this study were fundamentally accepting of food insecurity screening because of the perceived benefits that identification and support could bring. However, some women predicted difficulties if they chose to disclose, which highlights that, for some women, the challenges of experiencing food insecurity could be matched by the tension caused by disclosure.
3. *Preferences regarding food insecurity screening and support*

Participants offered insights into how the process of food insecurity screening could be conducted to optimise the patient's healthcare experience, to maintain their dignity and encourage disclosure in a safe and caring environment. This included views about screening modality, who should conduct screening and when and expected supportive strategies offered by the antenatal hospital.

In general, participants were comfortable with clinician‐facilitated, face‐to‐face screening. However, there was a suggestion that screening could occur via self‐administered assessment, specifically paper‐based, to avoid embarrassment.

I like the paper version. Just write on it and no one else can see what's on there. And then you can just fold it up and put it in a box. (22 years, first child, Australian)

For participants who were comfortable with clinician‐facilitated screening, it was imperative that clinicians approached screening in a nonjudgemental, sensitive manner and provided person‐centred care.

When you get some help, it should make you happy [and] proud. But when you get some help and you feel like, I wish this is not the way I get help … It makes you not someone who gets help, although you know that you want it. (33 years, fifth child, African)

Most participants did not have a preference about the screening clinician's occupation and reiterated that the more important factor was the sensitive, nonjudgemental approach to screening. Participants could foresee that clinicians who had already demonstrated a caring nature would be their preference.

I would trust that question from someone who knows me a lot … I've now developed a relationship with my midwife, she's really lovely. But if it were a random person who might be really rushed, or I'd never met before … I think that those contexts are so important. (33 years, first child, North American)

Other participants perceived that a sensitive approach could be taken by clinicians regardless of whether they were to provide continuous healthcare, for example, in the case of an initial assessment by a midwife at the first antenatal appointment. It seemed logical to participants to include food insecurity screening within a comprehensive assessment of key antenatal health indicators, such as mental health and family violence, usually conducted at the initial appointment.

I think it should be in that initial conversation, when they ask you privately about domestic violence, wellbeing and your stress level … because you're already asking the person confronting questions, and then you're just saying, can you also afford to eat food and have the right nutrition? (29 years, second child, South Asian)

Participants who were comfortable with clinician‐facilitated screening also provided suggestions for multimodal screening, with the presumption that not all pregnant women would feel comfortable with face‐to‐face screening. Suggestions included private mobile phone messaging via SMS, QR codes at the pregnancy clinic linking to an online screening survey and the option to complete screening via the ‘patient portal’ functionality of an electronic medical record.

Participants also had strong views about subsequent supportive strategies that could be offered by antenatal clinicians, and universally believed that the hospital was well placed to provide support. Such was the solutions‐based view regarding the ultimate purpose of screening that one participant stated that it was pointless to enquire about food insecurity if there was nothing the clinician could offer in support. Some participants suggested that a list of potential support strategies could be provided to all pregnant women at their first visit to an antenatal healthcare setting, before screening even occurring, to pre‐emptively prepare the women for the experience of screening. In another suggestion to reduce the stigma associated with accessing food security support, one participant suggested that the clinician could write a referral letter for pregnant women to present to their chosen support service, rather than be forced to articulate her need. The range of supportive strategies suggested by participants was indicative of the Australian context in which there is no established government‐led nutrition assistance programme for pregnant women. Strategies included referral to internal support services, referral to external support services such as food pantries, provision of free multivitamins, grocery vouchers, fruit and vegetable boxes delivered to home and education on healthy eating within a budget. Table 2 describes participants' suggestions.

**Table 2:** Supportive strategies suggested by participants.

Supportive strategy	Exemplar quote
Referral to support services (internal and external)	‘It'd be nice … if they offered me, would you be keen on going to a dietitian? This might help with your thyroid and help with your iron. I would love for someone to have told me that with both my pregnancies, instead of me having to outsource this out of my own pocket’.
	‘So if you could offer them supports from other services [like] the Salvation Army … they give you food parcels, stuff like that. Helping them get organized with services like that to help with food supply … that would help’.
Vouchers for groceries and nutrition supplements	‘I feel like the first place to start are little vouchers … if you are in financial hardship and you don't want to tell anyone, you're going to take a voucher and you don't have to tell anyone about that’.
	‘…supplements, maybe Elevit, offer any supplements or maybe, I think they mentioned Sustagen or something’.
Food packs or hampers	‘…have food packs [in the clinic waiting room] … that's got everything that you need. There's a piece of fruit, there's dairy, like milk or yogurt and sandwich or a muesli bar, that you could get at your appointments. And I know that doesn't fix a whole problem, but even one meal when someone might be struggling to get three on the table could be helpful. A volunteer [could] come around and kind of offer it’.
	‘…food delivery, maybe it might be weekly, fortnight, I don't know how. It will really help them at least weekly if they will be able to until they deliver that baby. Wherever they are located so they can get deliveries with the food that they select or the food that they want’.
Education for meal planning on a budget	‘I think some kind of access to education, whether that be classes or online or I don't know what it would be, could be helpful if people were open to that’.

## 4 DISCUSSION

This is the first qualitative study to explore the views and preferences of Australian food‐insecure pregnant women regarding food insecurity screening and support within an antenatal healthcare setting. Women in this study were accepting of food insecurity screening being conducted as part of their routine healthcare and endorsed having a range of supportive strategies offered to them if required. Women displayed a pragmatic attitude towards screening and identified potential benefits, such as feeling supported by their clinician to have a healthy pregnancy, and reduced pressure to voluntarily articulate their hardship. The women in this study gave suggestions for the implementation of food insecurity screening in antenatal healthcare, to optimise their healthcare experience, maintain their dignity and feel able to disclose within a safe and caring environment. These results indicate that universal food insecurity screening and support in the antenatal setting is likely to have support from pregnant women and should be considered in the reorientation of healthcare delivery to include this practice within routine care. Identifying and responding to food insecurity during pregnancy is a key step in laying a foundation that promotes optimal nutrition and health for all pregnant women and their offspring and is urgently needed to help mitigate adverse maternal and child health outcomes associated with poor antenatal nutrition.

Women in this study perceived food insecurity screening to be more acceptable during pregnancy than at other life stages. Women's understanding about the importance of antenatal nutrition and their perception of maternal responsibility to nourish their child in utero contributed to this viewpoint. This finding highlights an enabling factor towards food insecurity disclosure that may influence antenatal clinicians to engage in routine screening. It also shows that pregnancy could be an important time to initially identify food insecurity within a household, given that women may be more likely to disclose at this time for the sake of a healthy pregnancy. Other research in the United States and Australia has shown that food‐insecure, nonpregnant mothers often sacrifice the nutritional adequacy of their own diet for the sake of their children.^16^, ^58^, ^67^ This strategy of intrahousehold food allocation, among other coping strategies to manage the household food supply, may lead nonpregnant mothers to perceive that their food insecurity is manageable and not serious enough for disclosure to healthcare providers.^58^ Our study suggests that this may not be true for pregnant women, who hold an alternative view to disclosure that could be driven by their maternal identity. Although pregnant women are not a homogeneous group of ‘unilateral devoted nurturers’,^68^ it is important to consider that pregnancy may represent a window of opportunity to identify and address food insecurity, highlighting the need for screening to be embedded in routine antenatal healthcare.

When considering the operationalisation of screening, women in this study expressed comfort with clinician‐led, in‐person assessment. However, their approval was conditional on the sensitivity shown by the clinicians they would encounter when disclosing their food insecurity. The importance of clinicians' compassion to encourage women's disclosure of sensitive information during antenatal healthcare has been reported in other studies. For instance, a synthesis of qualitative research into the experiences of help‐seeking for perinatal psychological distress emphasised the importance of a clinician's nonjudgemental approach.^69^ Women included in the systematic review valued discussing their concerns with a clinician who seemed genuinely interested in their well‐being, who did not seem too rushed and who was familiar to them,^69^ all findings that concur with the views held by women in our study. Several studies to determine reasons for pregnant women to disclose intimate partner violence (IPV) have indicated similar findings relating to the clinician's caring approach.^70^, ^71^, ^72^ Spangaro et al.^70^ used qualitative configurational analysis to model pathways of pregnant women's nondisclosure of IPV to antenatal clinicians. The key conditions for nondisclosure included a sense of feeling uncared for by the screening clinician, demonstrated by their apparent discomfort and closed body language, lack of explanation and framing the purpose of screening and reading questions off the computer screen.^70^ The synthesis of evidence that encompasses antenatal screening of other sensitive topics such as IPV suggests that the approach taken by the screening clinician is of particular importance to decisions of disclosure. Findings in these fields point to the significance of connection with the clinician, for pregnant women to feel empowered to disclose.^73^ Further research into pathways to disclosure and nondisclosure of food insecurity to clinicians could provide more insight into training programmes for clinicians to establish a safe environment for pregnant women to disclose.

Beyond screening comes the important action of managing food insecurity within an antenatal healthcare setting. This is particularly important for Australia, where there is no government‐led nutrition assistance programme to support pregnant women, placing more pressure on individual antenatal healthcare settings to respond. Women in this study endorsed the opportunity to be offered support by clinicians to address food insecurity, viewing this as a logical follow‐up to screening and an expected component of clinical care. Supportive strategies suggested by women ranged from grocery vouchers to food packages; referral to external food aid organisations was also welcomed. One previous study has reported high participant satisfaction with a healthcare‐based intervention to address food insecurity during pregnancy. In this small, quasi‐experimental study, Fitzhugh et al.^74^ offered an emergency food package to pregnant women who answered affirmatively to the Health Vital Sign, a validated two‐item food insecurity screening tool, as well as a third item that aimed to identify urgent need for food. Survey‐measured participant satisfaction was ‘positive’, with over 71% of participants rating their healthcare experience as ‘extremely’ satisfactory.^74^ Further research into the efficacy and acceptability of healthcare‐based interventions is needed to ensure that the needs of food‐insecure pregnant women can be met within the resource limitations of antenatal clinics. Given the potential challenges that some women may face if they choose to disclose, such as domestic tension or violence, the types of supportive strategies are important to consider for women's safety and dignity. Therefore, supportive strategies should include discreet options, like grocery vouchers and nutrient supplements, for women's private access to avoid potential conflict at home. Hybrid effectiveness–implementation studies would be appropriate to explore food insecurity interventions during pregnancy and have previously been conducted in nonpregnancy healthcare settings.^75^, ^76^

### 4.1 Limitations

Despite these clear and important study findings, there are limitations to be considered. Selection bias is a limitation, as participants who were unwilling to or uncomfortable with being interviewed may have alternative views towards screening and support compared to the women who self‐selected to participate. Furthermore, this study was confined to one metropolitan hospital; women attending rural hospitals or other healthcare settings were not included. Future research should investigate the views of food‐insecure pregnant women living in rural and remote areas to complement these study findings, given the additional barriers to obtaining food in rural versus urban areas.^77^ A key strength of the present study is participant diversity, including migrant women, which is an important consideration as migrants and culturally diverse individuals experience food insecurity at disproportionate rates.^78^

## 5 CONCLUSION

The findings of this study that food‐insecure pregnant women endorse routine food insecurity screening and support is important for the delivery of antenatal healthcare to address this health issue. This study provides understanding about the preferences of food‐insecure pregnant women regarding food insecurity screening and support, given the unique circumstances of pregnancy that may influence disclosure and acceptance of help. Food insecurity screening and support could be embedded within routine antenatal healthcare, with care taken to maintain patients' dignity for safe disclosure within a caring environment. Specifically targeting antenatal screening will leverage the multiple clinical encounters to identify food insecurity and provide timely intervention, positively impacting pregnancy outcomes for women and children.

## AUTHOR CONTRIBUTIONS

**Julia Zinga**: Conceptualisation; investigation; writing—original draft; formal analysis; methodology; data curation. **Paige Pligt**: Conceptualisation; methodology; writing—review and editing; supervision. **Fiona H. McKay**: Conceptualisation; writing—review and editing; methodology; supervision.

## CONFLICT OF INTEREST STATEMENT

The authors declare no conflicts of interest.

## ETHICS STATEMENT

Ethics approval was granted by Royal Women's Hospital HREC (02773/RWH‐22‐33) and Deakin University HREC (2023‐016).

## Supporting information

Supporting information.

## Data Availability

The data that support the findings of this study are available from the corresponding author upon reasonable request.
