# Supplementary material for: Views and preferences of food‐insecure pregnant women regarding food insecurity screening and support within routine antenatal care
Source: Health Expect. 2024 Jan 7;27(1):e13956. doi: 10.1111/hex.13956 (PMC10771804; doi:10.1111/hex.13956)
Supplement: Supplementary file 1 — Supporting information. [file HEX-27-e13956-s001.docx]

**Supplementary material**: Interview guide. Views and preferences of food insecure pregnant women regarding food insecurity screening and support.

| Topic | Main question | Probes |
| --- | --- | --- |
| Pregnancy diet & affording | During your current pregnancy, what has been your experience with managing your household budget so that you could afford the food that you want to eat? | *Have there been times during this pregnancy where you have worried about having enough food, or when you have run out of food or money?  *Has the rising cost of living made it harder for you to make ends meet? *How?  *What strategies do you use to get by, so that you have enough food during your pregnancy? Such as food aid, or accepting food from family or friends? |
|  | Apart from being able to **afford** the food you want to eat, what has been your experience this pregnancy with **accessing** your food, for example, getting to and from the shops, and being able to prepare your meals? |  |
| Being screened during pregnancy | Now I am hoping to ask some questions about your expectations of the care you should receive from healthcare workers at xx hospital, like doctors and midwives. We are considering whether to check if all pregnant women that attend xx can access the food they want to eat. The assessment questions will be:   - “I worried whether my/our food would run out before I/we got money to buy more.” - “I/we couldn’t afford to eat balanced meals.”   Please indicate whether the statement was **often true, sometimes true, or never true** for you/your household in the last 12 months. | |
|  | How did you feel when you answered these questions? | Did you find them confronting? A relief? Some people think that this is a sensitive topic and that sometimes being asked these questions makes people feel a certain way… did that happen to you? How so? |
|  | Was there a specific question that made you feel that way? | What made you feel that way about that question? Could the question be worded differently? |
|  | Have you had conversations in the past with any healthcare worker about challenges in affording or accessing enough food? | How was this experience for you? How about prior to your pregnancy? Or regarding any of your personal concerns (not just about food)?  What about the situation made you feel comfortable / relaxed / uncomfortable?  What did you wish was different about that situation? |
|  | When it comes to receiving care at xx, do you **expect** to be asked by healthcare workers whether you are able to access and afford the food you need during your pregnancy? | Would this fit in with the care you have received from xx, or expect to receive? |
|  | Do you think healthcare workers **should** help patients with this issue? | Why / why not? |
|  | Do you think healthcare workers **can** help with this issue | Why / why not? |
|  | What, if anything, could be reasons to **not** tell a healthcare worker about your ability to access enough food? | What could make it difficult to talk to your healthcare worker about your household food needs? |
|  | What do you wish healthcare workers knew about the experience of having limited access to enough food during pregnancy? | What could make it easier to talk to your healthcare worker about this?  How can this experience be more comfortable? |
| Screening operationalisation | **Now I would like to shift to find out your feelings about HOW these questions could be asked.** | |
|  | Do you have a preference for the type of health worker that you would be most comfortable with to discuss your challenges accessing and affording enough food? | Prompt with types of available health workers if needed |
|  | Would it matter if these questions were asked in person, or if they were on a form that you complete on your own? | Why? |
|  | If you prefer to answer these questions in private, when would be the best time to answer these questions? | Eg. before your visit, during your visit, after your visit, at home vs hospital, or you don’t mind? |
|  | Preference to answer these questions on paper or on your phone via an electronic link sent by the hospital? |  |
|  | How comfortable would you be having other people on your healthcare team see information about your ability to access and afford enough food? | Who would you want to see the information vs not see? Why is this?  Privacy concern? Relevancy to main health issue / reason for attending hospital? |
|  | How frequently do you think these questions should be asked during pregnancy? |  |
| Support offered | If you were to be told by a healthcare worker that your responses to the screening questions indicated you were experiencing food insecurity, how would you want to be informed? | Verbally? electronic response if done via online link? How should it be worded, if verbal? |
|  | What do you think healthcare workers should do with information about patients’ social and economic needs? | Should healthcare workers try to connect patients to support services for food assistance? Give information to patients? Or is it enough that healthcare workers are just aware of your situation and how this may impact on your health? |
|  | How do you think xx hospital could support patients who are facing challenges to access or afford enough food? What could this look like? |  |
| **Do you have any other comments to make about this topic? Thank you for your time today.** | | |
